# Tracking real-time proximity in daily life: A new tool to examine social interactions

**DOI:** 10.3758/s13428-024-02432-1

**Published:** 2024-04-29

**Authors:** Loes H. C. Janssen, Bart Verkuil, Andre Nedderhoff, Lisanne A. E. M. van Houtum, Mirjam C. M. Wever, Bernet M. Elzinga

**Affiliations:** 1 Department of Clinical Psychology, Faculty of Social and Behavioral Science, Leiden University, Wassenaarseweg 52, 2300 AK Leiden, The Netherlands; 2 Leiden Institute for Brain and Cognition (LIBC), Leiden, the Netherlands

**Keywords:** Proximity, Ecological momentary assessment, Bluetooth beacon, Parent–adolescent interaction, Daily life

## Abstract

Social interactions, spending time together, and relationships are important for individuals’ well-being, with people feeling happier when they spend more time with others. So far, most information about the frequency and duration of spending time together is based on self-report questionnaires. Although recent technological innovations have stimulated the development of objective approaches for measuring physical proximity in humans in everyday life, these methods still have substantial limitations. Here we present a novel method, using Bluetooth low-energy beacons and a smartphone application, to measure the frequency and duration of dyads being in close proximity in daily life. This method can also be used to link the frequency and duration of proximity to the quality of interactions, by using proximity-triggered questionnaires. We examined the use of this novel method by exploring proximity patterns of family interactions among 233 participants (77 Dutch families, with 77 adolescents [*M*_age_ = 15.9] and 145 parents [*M*_age_ = 48.9]) for 14 consecutive days. Overall, proximity-based analyses indicated that adolescents were more often and longer in proximity to mothers than to fathers, with large differences between families in frequency and duration. Proximity-triggered evaluations of the interactions and parenting behavior were generally positive for both fathers and mothers. This innovative method is a promising tool that can be broadly used in other social contexts to yield new and more detailed insights into social proximity in daily life.

**Supplementary Information:**

The online version contains supplementary material available at 10.3758/s13428-024-02432-1.

## Introduction

Interpersonal relationships and social connectedness are of fundamental importance for human development and physical and mental health throughout the lifespan (e.g., Umberson & Karas Montez, 2010). Generally, people who spend more time interacting with others and have deeper interactions feel happier (e.g., Sun et al., 2020). In infancy, close relationships with caregivers develop first as a means to promote the proximity and safety of the infant (Bowlby, 1969; Bowlby, 1982), and the parent–child relationship remains one of the most proximal and important relationships for development and well-being even during adolescence (Bronfenbrenner, 1979; Sameroff, 2000). Time spent together between adolescents and parents has been shown to relate to adolescent well-being as well as to the quality of the parent–adolescent relationship (e.g., Dubas & Gerris, 2002; Desha et al., 2011; Offer, 2013). For instance, spending more time with parents has been associated with lower levels of depressive symptoms in adolescents (Desha et al., 2011; Manczak et al., 2019) and predicted decreases in family conflict over time (Dubas & Gerris, 2002). Also in intimate relationships, time spent with a romantic partner has been found to be related to higher levels of well-being (Hudson et al., 2020). However, spending (too much) time together can also result in tension and stress for adolescents and parents, for instance during the COVID-19 pandemic (Stänicke et al., 2023; Wiemer & Clarkson, 2022). Given its central importance for well-being, it is pivotal that we can assess time spent together in a reliable and valid manner. In this paper we describe a new method, in the hope that this will aid the field in furthering our insights into the impact of social proximity in daily life.

So far, assessments of time spent together and dyadic interactions are mostly based on questionnaires - either retrospectively or more recently, also in daily life (e.g., Dubas & Gerris, 2002; Desha et al., 2011; Offer, 2013). Even though these subjective reports have substantially improved our understanding of social proximity, these assessments are prone to reporter bias and only generate a rough estimation of time spent together, and hence do not provide accurate, objective data on the amount of time people spend together over the course of a day or week. In animal research, it is rather common practice to track the frequency and duration of social behavior by using proximity or other objective measures such as radio trackers (e.g., Hunt et al., 2012). Although researchers started to use similar methods as research tools in our own species (e.g., Salo et al., 2021), these methods are still limited to for instance small-scale settings and research settings. In sum, there is a clear need for more detailed information on *objective* behavioral patterns of dyads’ proximity throughout the day or week in daily life. This may generate important additional insights into social proximity between individuals in daily life, be it parents and children, partners, or friends. In the current study, we therefore introduce a novel and more detailed method to passively track proximity between dyads every 5 minutes and assess the frequency and duration of being close to each other in the daily flow of life. As an illustration, we focus on the proximity between adolescents and parents. With the use of Bluetooth low energy (BLE) beacons and a smartphone application, we aimed to *observe* detailed real-time adolescent–parent proximity in the natural context of the family and assess the quality of these interactions between adolescents and parents by triggering brief questionnaires based on this proximity (Gupte & Eliassi-Rad, 2012).

### Bluetooth proximity tracking

Proximity between people can be detected by several passive sensors (Wi-Fi, GPS, or Bluetooth) that are currently available on almost all smartphones. GPS and Wi-Fi are not that specific, with accuracy ranging between 3 and 50 meters. Moreover, GPS is based on satellites and signals worsen near buildings and are too weak indoors. The use of Bluetooth seems most promising in providing ecologically valid data as it can measure the proximity of people with an accuracy of 0 to 5 meters even indoors, depending on the settings of the device (Liu & Striegel, 2011). Recently, researchers in the field of social sciences have started to test different approaches for using Bluetooth as a method to track proximity and assess social networks or dyadic proximity. Broadly three different methodological approaches can be distinguished. A first approach is detecting proximity between persons by only using (wearable) Bluetooth devices such as ActiGraph accelerometers that can either send or receive a Bluetooth signal, but not both. Studies showed that this approach is valid and reliable in a controlled and real-life setting both indoors and outdoors (Dlugonski et al., 2019; Kuzik & Carson, 2018). In a second approach, participants are provided with a research smartphone that detects the proximity of others’ phones or BLE beacons. Detecting proximity between dyads or larger networks with this approach is also promising and feasible (Maharjan et al., 2021; Van Woudenberg et al., 2020). The third approach involves installing an application on participants’ own smartphones. One previous study piloted and tested an intervention for expressing gratitude, using proximity to other persons (i.e., social proximity) to trigger notifications (Ghandeharioun et al., 2016), and another study showed that proximity registered by sociometric badges was more strongly related to self-report than when registered by a designed smartphone app (Boonstra et al., 2017).

These studies have shown that proximity between persons can be tracked using smartphone Bluetooth with or without BLE beacons. The majority of studies, however, included small sample sizes (up to 40 participants) and the few studies that included larger samples in real-life settings used a research smartphone (e.g., Stopczynski & Lehmann, 2018; Stopczynski et al., 2014; Van Woudenberg et al., 2020). While this has certain advantages (e.g., similar phone type and up-to-date software), it may also be burdensome for participants to carry two smartphones throughout the day. Moreover, when only using Bluetooth of the smartphone, combining different smartphone operating systems (i.e., iOS and Android) to track proximity can be complicated. It is not yet possible for an iOS device to detect and pair with an Android device over Bluetooth. Solely using Bluetooth of the smartphone, without additional use of a Bluetooth beacon, would therefore limit the usability of the method. The current study addresses these limitations by using a novel method to unobtrusively assess patterns of proximity between adolescents and parents (i.e., frequency and duration) in their daily lives with the use of BLE beacons combined with an application installed on their own smartphones. Additionally, the current study combines this approach with triggering questionnaires based on this proximity, which has not been done before.

### Proximity as indicator of parent–adolescent interactions

Due to the common availability of smartphones, the use of ecological momentary assessment (EMA; Stone & Shiffman, 1994) nowadays enables assessing subjective experiences of interactions in a more ecologically valid way in daily life (Trull & Ebner-Priemer, 2009) with reduced recall bias (Schwarz, 2007). This fairly new line of work can enhance our understanding of the dynamic interactions between, for example, adolescents and parents (Keijsers et al., 2021; Janssen et al., 2021). However, this method is not without limitations. Most notably, impactful interactions can be missed when random sampling schemes are used (i.e., questionnaires triggered randomly throughout the day), whereas instructing families to indicate themselves when they had a “meaningful” interaction (i.e., event-contingent sampling) may be prone to bias. Especially when interactions are either very positive or heated or unpleasant, participants may not think about or feel like reporting this. Using questionnaires triggered by physical proximity as tracked by our novel method can overcome these limitations. To gain more insight into whether frequency and duration of proximity were related to adolescents’ and parents’ subjective experiences of interactions, such as the pleasantness of the interaction and affect states, we therefore used proximity-triggered questionnaires that were sent shortly after an adolescent and parent had been in proximity for more than ten minutes.

### The current study

The primary aim of the current study was to provide an illustration of a novel method to assess patterns of physical proximity (i.e., frequency and duration) between persons with BLE beacons and a smartphone application in the natural context. Since parent–child interactions are crucial for well-being (Bronfenbrenner, 1979; Sameroff, 2000), we focused here on the proximity between adolescents and parents, but this method can also be applied as a meaningful tool in other social domains (e.g., tracking the proximity of romantic partners, friends, or colleagues).

Since previous self-report studies reported that mothers spent more time with adolescents than fathers (Larson & Richards, 1991; Phares et al., 2009; Van Lissa & Keizer, 2020), we expected to find similar patterns based on our objective assessments. Moreover, we assessed the quality of the interactions using proximity-triggered questionnaires and explored whether quantitative aspects of being in proximity (e.g., frequency and duration) are related to the subjective experiences of these interactions, such as the pleasantness of the interactions.

## Methods

### Sample

A subsample was used from RE-PAIR (Relations and Emotions in Parent Adolescent Interaction Research), a Dutch multimethod two-generation study examining the bidirectional interplay between parent–child interactions and adolescent mental well-being including adolescents and their parents. The RE-PAIR study consisted of four parts: online questionnaires, a research day at the lab, two weeks of EMA, and a functional magnetic resonance imaging (MRI) scan session with the adolescent and one parent. The RE-PAIR study was approved by the Medical Ethics Review Committee (METC) of Leiden University Medical Centre (LUMC; research protocol: P17.241). The subsample in the current study included families with an adolescent without psychopathology and focused on the EMA part of RE-PAIR.

#### Inclusion

Families were included in the study if adolescents were aged between 11 and 17 years, started secondary school, lived with at least one primary caregiver who wanted to participate, and all had a good command of the Dutch language. Participation with two parents - if possible - was preferred but this was not a requirement. Families were excluded if adolescents had a current mental disorder, a history of major depressive disorder (MDD) or dysthymia, or a history of psychopathology in the last two years. Adolescent psychopathology was assessed on the research day during a face-to-face semi-structured interview, the Kiddie-Schedule for Affective Disorders and Schizophrenia–Present and Lifetime Version (K-SADS-PL; Reichart et al., 2000). Adoptive, foster, and stepparents (*n* = 14) were allowed to participate if they were involved in the upbringing of the adolescent for at least five years and if adolescents perceived the parent as a primary caregiver. For reasons of clarity, they will be referred to as mothers and fathers from here onwards. Owning a smartphone was not an inclusion criterion but was necessary for the EMA part of RE-PAIR. All family members who participated had a smartphone.

For a detailed description of the recruitment procedure see (Janssen et al., 2021). The final sample of RE-PAIR consisted of 80 families with a total of 233 participants (80 adolescents, 153 parents). Two fathers (1.3% of parents) did not participate in the EMA part of RE-PAIR due to too much time investment, resulting in a final sample for the EMA of 231 participants (80 adolescents, 151 parents). Since the BLE beacon cards did not work in three families (3.8% of families), the final sample for the current study consisted of 77 families (77 adolescents, 145 parents). The majority of adolescents (97.4%) and parents (94.5%) were born in the Netherlands. For detailed information on missing data see Appendix 1. Sample demographics are presented in Table 1.

**Table 1:** Sample demographics

Variables	*N*	
Adolescents		
Gender, % Female, (*n)*	77	64.9 (50)
Age (years), *M (SD)*^a^	77	15.9 (1.38)
Highest level of education, % (*n*)	77	
Vocational education		13.0 (10)
Advanced secondary education		33.8 (26)
Pre-university education		44.2 (34)
Secondary vocational education		6.5 (5)
Higher professional education		2.6 (2)
Living situation	77	
With biological mother		6.5 (5)
With biological mother and father		77.9 (60)
Other^b^		15.6 (12)
Daily positive affect^c^, *M (SD)*	77	5.47 (0.76)
Daily negative affect^c^, *M (SD)*	77	1.51 (0.63)
Parental warmth—mother^c^, *M (SD)*	76	5.88 (0.81)
Parental warmth—father^c^, *M (SD)*	69	5.76 (0.99)
Parental criticism—mother^c^, *M (SD)*	76	2.03 (1.00)
Parental criticism—father^c^, *M (SD)*	69	1.86 (0.92)
Parents		
Gender, % Female, (*n)*	145	52.4 (76)
Age (years), *M (SD)*^a^	145	48.9 (5.93)
Highest level of education, % (*n*)	145	
No diploma		0.7 (1)
Lower vocational education		7.6 (11)
Intermediate vocational education		26.2 (38)
Higher vocational education or scientific education (university)		65.5 (95)
Parental warmth—mother^c^, *M (SD)*	76	5.68 (0.69)
Parental warmth—father^c^, *M (SD)*	76	5.38 (0.73)
Parental criticism—mother^c^, *M (SD)*	69	2.45 (0.95)
Parental criticism—father^c^, *M (SD)*	69	2.47 (0.91)

^a^Age at research day

^b^This included parent and stepparent, alternating between father and mother, or living with adoptive/foster parents

^c^Person mean

### Procedure

Families were recruited via public places and (online) social media. Interested families were briefed about the study and were screened via a telephone call. If all inclusion and no exclusion criteria were met, an appointment was scheduled for a research day in Leiden. Adolescents and their parents provided written active informed consent on the research day. For adolescents younger than 16 years of age, parents with legal custody also signed informed consent for the adolescent. Adolescents and parents received face-to-face instructions during the research day about the EMA procedure, proximity tracking, and proximity-triggered questionnaires. Next, researchers assisted participants with installing the Ethica Data application (Ethica Data, n.d.) on their smartphones for the EMA and each family member received a personal BLE beacon for proximity tracking. Each family member also received written instructions and their individual account information for the Ethica app. Participants were instructed to keep the BLE beacon (the size of a credit card) in their own phone case throughout the EMA period (14 consecutive days) or in the sticky card holder case provided by the researchers. Participants were additionally asked to carry their smartphone with them as much as possible, also inside their homes. A power bank was offered to participants if the battery life of their phones was impaired. Generally, the EMA started the next Monday after the research day; however, in the case of holidays and exam weeks for adolescents, EMA started the first Monday thereafter. In addition to proximity tracking and proximity-triggered questionnaires, participants received four semi-random scheduled EMA questionnaires a day (see Janssen et al., 2021 for detailed information).

Researchers monitored proximity tracking and proximity-triggered questionnaires by checking real-time data in Ethica on a daily basis and were available for questions or problems via WhatsApp, telephone, and email. If problems arose with proximity tracking or participants reported not receiving proximity-triggered questionnaires, researchers inspected available proximity data and logs via the Ethica dashboard. Participants were asked to check and possibly change settings. On the last day of the EMA, a message was sent to thank participants and remind them of the scheduled phone call after the EMA to evaluate the EMA and to remind them to send the BLE beacons back to the research team. This part of the RE-PAIR study, assessing EMA data among adolescents without psychopathology and their parents, was conducted during the period between September 2018 and November 2019. As compensation for EMA, parents received €20, and adolescents €10.

### Equipment and measures

#### Proximity tracking

The Ethica application is a platform that can be downloaded on smartphones to allow participants to complete questionnaires on their own phones. A dashboard is available online for researchers to design their questionnaires, enroll participants, keep track of compliance, access real-time visualizations of the data, and export the data. In response to our request, Ethica developed the feature to combine tracking proximity via BLE cards and sending questionnaires with their application, which was not yet possible within Ethica or any other application when we were designing the study (2017/2018). Answering questionnaires in the Ethica app, including the proximity-triggered questionnaires, was not dependent on internet connection, as the app also works offline. During the development and pilot of the feature, Ethica tested several BLE beacons from different companies. The combination of the Ethica app with BLE beacons of Kontakt.io (https://kontakt.io/) seemed to work best. At that time, Kontakt had several types of BLE beacons available (e.g., cards, wristbands, buttons), which differed with regard to for instance battery life and transmission power. For our study aim, it was important that BLE cards were easy to carry around near the phone with a long battery life. Therefore, we selected the Kontakt BLE Card Tags CT16-2 (i.e., BLE beacons). A beacon broadcasts a signal that can be detected and the transmission power of the beacon determines how powerful the signal is transmitted. The BLE beacon cards we selected were set to −20 dBm (decibel-milliwatts), called transmission power level 1, with an approximate proximity detection range of 4 meters or less (a smaller range was not possible with this specific type of beacon but the range fitted our aim). BLE beacons of Kontakt have eight power levels (ranging from 0 to 7) and the higher the level, the more powerful the signal and the wider the range. As we aimed to focus on proximity between adolescents and parents (also being in the same living room) and possible parent–adolescent interactions, a transmission power setting of 1 seemed most suited. Piloting showed that walls blocked proximity tracking.

The Ethica app scanned for BLE beacons in proximity. The iBeacon profile of the BLE beacons was used, which has three adjustable parameters: universally unique identifier (UUID), major value, and minor value. The UUID was study-specific, and the Ethica app collected data on broadcasts of BLE beacons that corresponded to the study UUID. The major and minor values were used to identify BLE beacons with greater accuracy, with the major value identifying BLE beacons of a family and the minor value distinguishing individual BLE beacons within a family. The BLE beacons in the current study were reused and the major and minor value parameters were changed accordingly. Due to smartphone manufacturer constraints scanning took place approximately every 5 minutes. Proximity data were logged by the Ethica app when at least one family member was carrying their smartphone (with the Ethica app installed on it) and another family member was carrying their BLE beacon and were close to each other within the specified range. Each smartphone scanned independently for BLE beacons. In order to scan for BLE beacons, the Ethica app had to be active (in the background), had to have permission to access location services, and Bluetooth had to be turned on. Turning off the smartphone, retracting permission to access location services, switching Bluetooth off, manually terminating the Ethica app (which happened mostly on iPhones since Android allows applications to remain active), using battery-saving mode, and using night or flight mode blocked the scanning process. If participants terminated the app manually or switched off Bluetooth, the Ethica app would show a notification that the app was terminated, asking participants to reopen the app or switch Bluetooth on.

Raw BLE beacon data on when the Ethica app on participants’ smartphones detected BLE beacons in proximity was exported from Ethica Data and loaded in R version 4.0.1 (R Core Team, 2020). These data include information on date and time, device ID, name detected BLE beacon card, Received Signal Strength Indicator (RSSI), and major and minor values. We added the start and end date of the participation period (14 consecutive days of EMA) and the type of smartphone of each individual to the data. A descriptive overview of connections per person was obtained and checked visually (e.g., whether the Ethica app on the adolescent’s smartphone of family A detected the BLE beacons of the mother and father of family A). As the proximity data collection was two-sided (i.e., proximity based on detecting the BLE beacon of the mother by the Ethica app on the smartphone of the adolescent and proximity based on the BLE beacon of the adolescent detected by the Ethica app on the smartphone of the mother), data for both sides were checked. If the data seemed incorrect (i.e., incorrect ID number, incorrect major or minor values, incorrect combination of major and minor), the specific data were further inspected to investigate the cause of the errors (e.g., if the Ethica app on adolescent’s smartphone of family A detected the BLE beacons of mother of family B). This inspection resulted in the following cleaning steps. First, any data related to test accounts in Ethica which were used in the pilot phase were removed. Second, data for excluded families, families who did not start EMA, and families of adolescents with depression were removed. Third, incorrect major and minor values were checked and manually corrected. These were most likely caused by reusing the BLE beacons, either by making a manual error when changing the BLE beacon settings or an error in synchronization of the BLE beacon settings. We found incorrect major and minor values in the data of 11 persons. If the error was retraceable, we manually corrected them. For example, adolescents of family A carried BLE beacon 1 in January 2019 with specific minor and major values. The settings of that BLE beacon were changed in order for the mother of family B to use it in March 2019. The equipment of the adolescent of family B detected BLE beacon 1 and data included the minor value of the adolescent of family A, but all other information (e.g., ID, major) referred to the mother of family B, and data was collected during March 2019. Fourth, data on connections to other BLE beacons outside the family or devices were removed from the dataset. For example, the Ethica app on smartphones of family B detected BLE beacons of family A due to having functional MRI (fMRI) scanning sessions on the same day, with family A returning their BLE beacons (after participation) while family B was still participating. Lastly, all data outside the participation period was removed from the dataset. BLE beacon data was collected continuously and some families received their instructions and BLE beacon cards day(s)/week(s) before the start of the EMA participation period. For detailed information on missing data see Appendix 1.

#### Proximity-triggered questionnaires

**Fig. 1:**
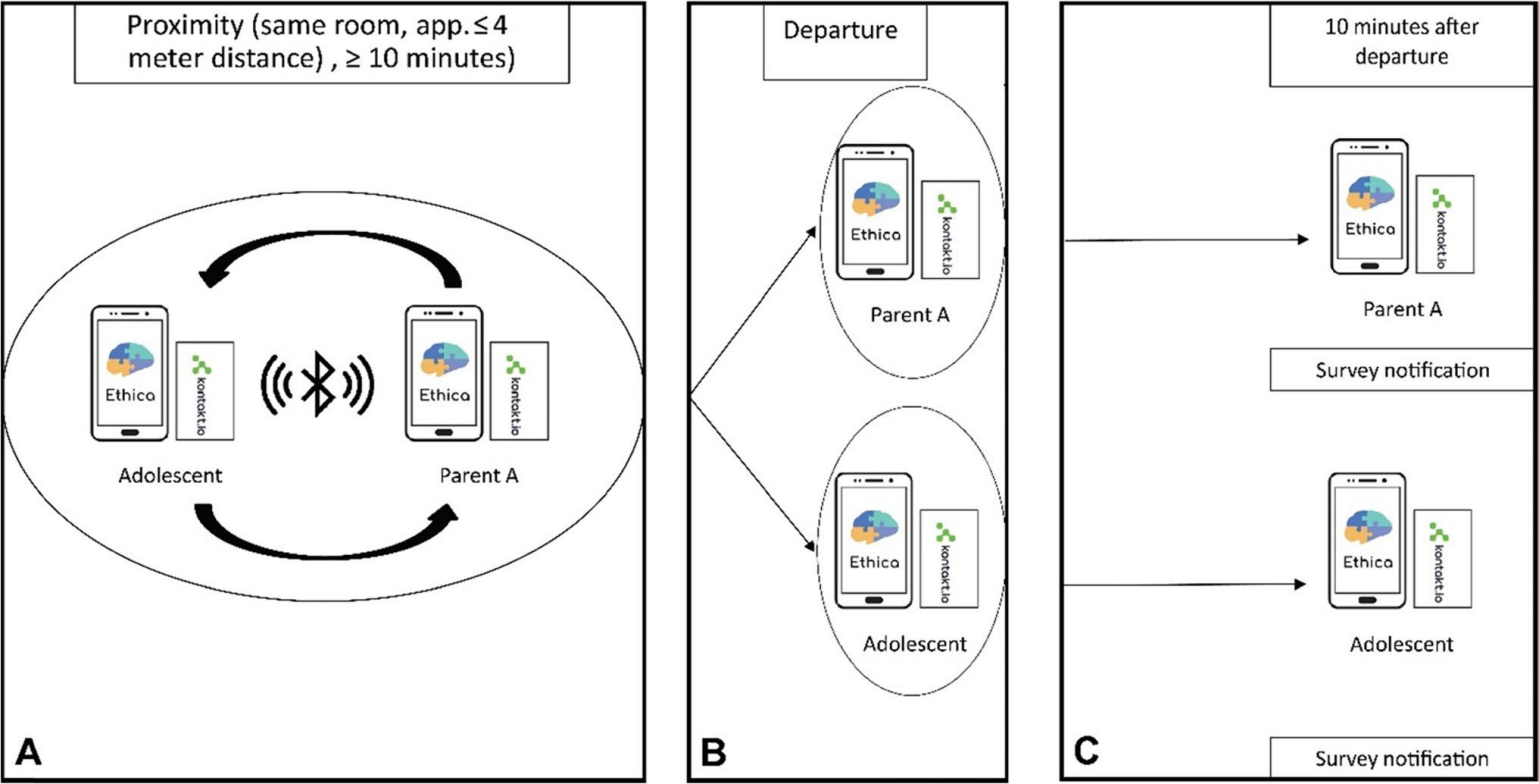
Graphical representation of proximity-triggered questionnaires. Panel **A** shows proximity tracking with an adolescent and one parent (with their phones and BLE beacons) being in the same room in proximity (i.e., within a range of approximately 4 meters distance or less). Adolescent and parent depart from each other (Panel **B**). If the adolescent and parent were in proximity for at least 10 minutes, they received a proximity-triggered questionnaire in Ethica 10 minutes after departure (Panel **C**)

Participants received questionnaires based on proximity tracking as described above. If adolescents and parents departed from each other, after being in proximity for at least 10 minutes, a proximity questionnaire was triggered 10 minutes after departure. These cutoffs (representing approximately two scanning intervals) were chosen to reduce triggering questionnaires when adolescents and parents were not in proximity (false positives). Adolescents received separate questionnaires regarding interactions with mothers and fathers and could thus receive two questionnaires after being in proximity to both mother and father. At first, the questionnaires expired after 10 minutes, but this was changed to 30 minutes after the participation of three families. If a proximity questionnaire was triggered, it was blocked for the next 4 hours to limit the potential number of questionnaires as participants also received four semirandom scheduled questionnaires. See Fig. 1 for a graphical presentation of the proximity tracking process to trigger questionnaires.

#### Frequency of proximity

The frequency of physical proximity between adolescents and parents during the day was calculated per dyad by counting the number of occurrences that either the Ethica app on the adolescent’s smartphone detected their parent’s BLE beacon or the parent’s smartphone detected their adolescent’s BLE beacon. If the smartphones of both the adolescent and parent detected each other’s BLE beacon around the same time (within a time interval of 2.5 minutes), it was counted as one occurrence (see Appendix 2 for details on the specified time interval of detecting each other’s BLE beacon).

#### Duration of proximity

Duration of time spent in proximity during the day (in minutes) was calculated when proximity was detected in two (or more) consecutive scanning intervals. Time intervals between the scans were summed when (i) the adolescent was in proximity of the parent for two or more scans, (ii) the parent was in proximity of the adolescent for two or more scans, and (iii) the adolescent and parent were in proximity of each other around the same time (within a time interval of 2.5 minutes) for two or more scans, using adolescents’ beacon data as the basis for the calculation of duration. Scanning behavior (approximately every 5 minutes) can be influenced by smartphone brand and result in irregular scanning behavior. To account for these irregularities, a wider range was used. To gain more insight into the range and distribution of time intervals between scans, we calculated the time (in minutes) between each scan per individual. Descriptive statistics indicated that although the median value was 5 minutes, there were substantial variations. Based on visual inspection of graph A3.2 (see Appendix 3 for figure and more details), it was decided to use a cutoff of a maximum of 7 minutes per scan.

#### Pleasantness of interaction

If a proximity questionnaire was triggered, adolescents and parents first indicated whether they had actually spoken to each other. If this was not the case, no follow-up questions were asked. If they had spoken to each other, this was counted as an interaction. They subsequently received questions about this interaction (i.e., pleasantness of interaction, affect, and parenting behavior). Adolescents and parents indicated the pleasantness of the interaction by answering the question “How was this contact?” on a seven-point Likert-type scale with answer categories ranging from 1 (*very annoying*) to 7 (*very nice*).

#### Affect

Adolescents and parents rated their own affect states during the interaction with an adapted and shortened five-item version of the Positive and Negative Affect Schedule for Children (PANAS-C; Ebesutani et al., 2012; Watson et al., 1988). Two positive affect states (happy and relaxed) and three negative affect states (sad, irritated, and guilty) were assessed by asking “How did you feel during this contact?” followed by “Happy,” “Relaxed,” “Sad,” “Irritated,” and “Guilty.” Answers were given on a seven-point Likert-type scale with answer categories ranging from 1 (*not at all*) to 7 (*very*). To create a score for positive affect per interaction, an average score of happy and relaxed was calculated for adolescents and parents separately. To create a score for negative affect per interaction, an average score of sad, irritated, and guilty was calculated for adolescents and parents separately.

#### Parenting

Adolescents rated the parenting behavior of their parent during the interaction by answering the questions “How well did your mother/father listen to you?”, “How well did your mother/father understand you?”, “How critical was your mother/father towards you?”, and “How dominant was your mother/father?”. Answers were given on a seven-point Likert-type scale with answer categories ranging from 1 (*not at all*) to 7 (*very*). Parents rated their parenting behavior during the interaction by answering the questions “How well did you listen to your child?”, “How well did you understand your child?”, “How critical were you towards your child?”, and “How dominant were you towards your child?”. Answers were given on a seven-point Likert-type scale with answer categories ranging from 1 (*not at all*) to 7 (*very*). Two subscales were created, parental warmth and parental criticism. An average of listening and understanding behavior per interaction was calculated for adolescents and parents separately to assess parental warmth. An average of critical and dominant behavior per interaction was calculated for adolescents and parents separately to assess parental criticism.

### Strategy for descriptive analyses

R version 4.0.1 (R Core Team, 2020) was used for the descriptive analyses. As the aim of the study was to illustrate the method, the focus was on between-person analyses. Frequency of proximity between adolescents and mothers and between adolescents and fathers during the day was calculated by counting the occurrences of being in proximity throughout the 14 days, on average per day, and on average per week and weekend day. Duration of proximity between adolescents and mothers and between adolescents and fathers during the day was calculated on average throughout the 14 days, on average per day, and the average duration of being in proximity per moment. Normal distribution and equality of variances were checked and when assumptions were not met, appropriate nonparametric tests were used to examine differences between adolescent–mother and adolescent–father dyads in frequency and duration. Moreover, we examined whether frequency and duration differed between adolescent girls and boys by using appropriate nonparametric tests and if these were related to age by using Pearson correlations. Next, we described the use of proximity-triggered questionnaires and adolescents’ and parents’ subjective experiences of parent–adolescent interactions (i.e., pleasantness of interactions). Lastly, to explore whether the frequency and duration measures were related to experienced pleasantness, parenting behavior, and adolescent affect, Pearson correlations were used.

## Results

Since some families reported that adolescents were not allowed to take their smartphones to their bedrooms during nighttime and smartphones were placed elsewhere, data collected during nighttime were removed from the dataset. The specification of nighttime was based on self-report EMA data of participants in RE-PAIR about bedtime and rise time from the morning questionnaires of the standardized trigger schedule (see Appendix 4 for rationale). Based on these restrictions and inspection of the data, it was decided to use the data collected from Monday through Friday between 7:00 AM and 9:30 PM as well as data collected on Saturday and Sunday between 9:00 AM and 11:00 PM, data outside this time interval was removed.

### Description of parent–adolescent proximity

#### Frequency of proximity

**Fig. 2:**
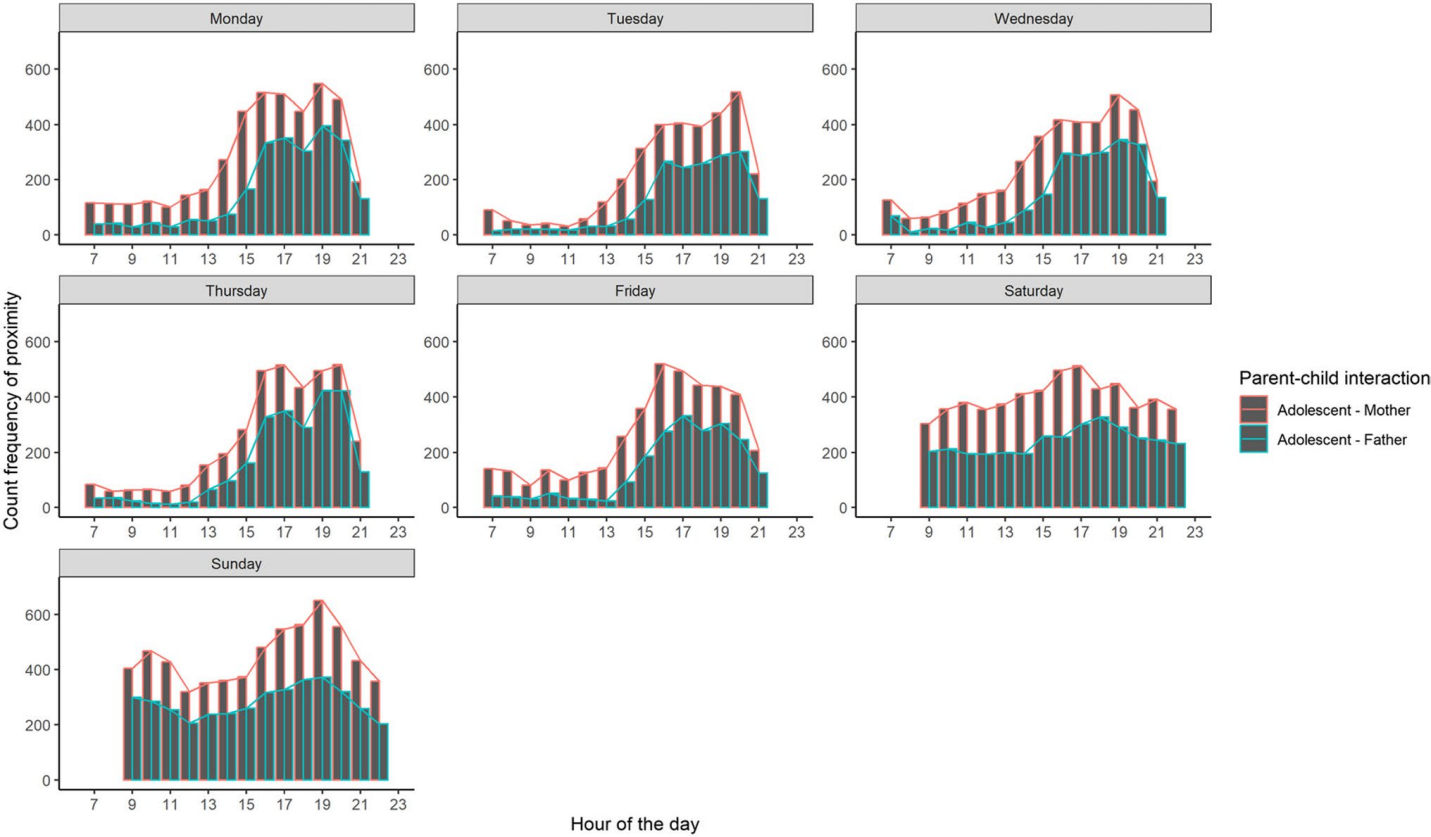
Frequency of proximity between adolescents and parents throughout days of the week per hour, separated for adolescent–mother and adolescent–father dyads, combining two weeks of data

**Fig. 3:**
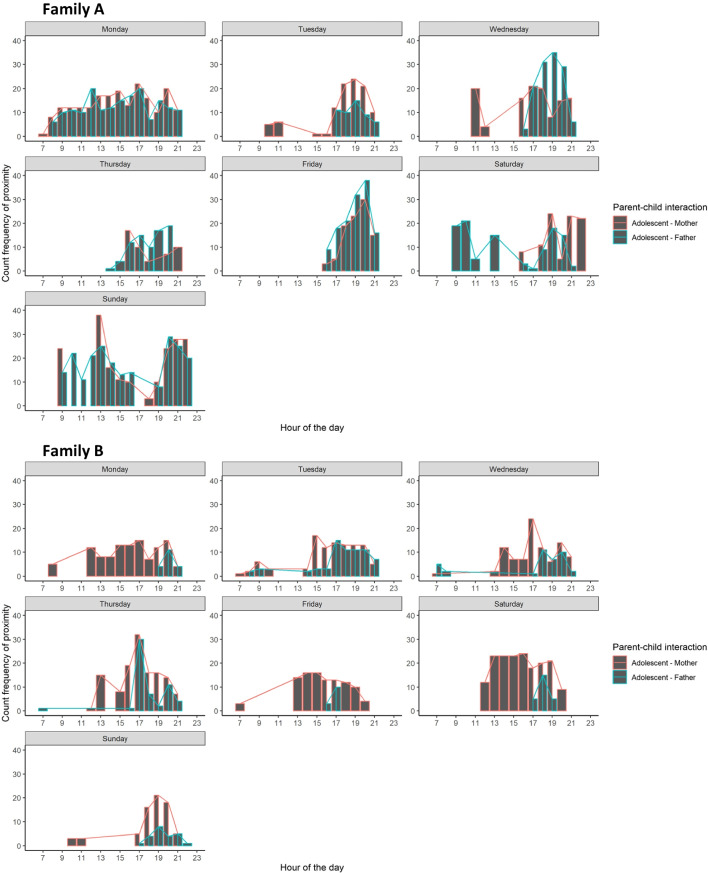
Frequency of proximity between adolescents and parents throughout days of the week per hour, separated for adolescent–mother, and adolescent–father dyads for family A and B, combining two weeks of data

Table 2 provides descriptive information on the average frequency of proximity between adolescents and parents throughout two weeks. To gain further insight into when adolescents and parents were together during the day, the frequency of being in proximity was plotted per hour throughout days of the week for the whole sample (see Fig. 2), and for two families, to illustrate the raw data and the variation between families (see Fig. 3). On average, on weekdays, proximity between adolescents and parents started to increase from 1:00 PM with a peak around 4:00 PM, followed by a short decrease and then a peak again around 7:00 PM or 8:00 PM. A different pattern can be observed during the weekend when adolescents and parents were more often in each other’s proximity throughout the day with a peak around 5:00 PM on Saturday between adolescents and mothers and around 6:00 PM between adolescents and fathers and around 7:00 PM on Sunday.

**Table 2:** Descriptive statistics of the frequency and duration of adolescent–parent proximity during the two weeks

					Paired Wilcoxon’s signed-rank test	
	*N*	*Mdn*	Min	Max	*z*	*p*
Frequency						
Adolescent–mother	75	334.00	41.00	1108.00		
Adolescent–father	68	232.50	9.00	893.00	−5.079^a^	< .001
Duration (in minutes)						
Adolescent–mother	75	823.00	104.26	3715.36		
Adolescent–father	67	508.38	54.14	3677.11	−5.019^b^	< .001

*Note.* The median was reported since the frequency and duration of proximity between adolescents and parents was non-normally distributed (all *p*s < .001)

^a^ *n* = 66

^b^ *n* = 65

Throughout the two weeks, adolescents were more often in proximity to mothers than to fathers (paired Wilcoxon’s signed-rank test: *z* = −5.079, *p* < .001). On average, proximity was detected 23 times per day between adolescents and their mothers (*Min* = 1, *Max* = 199) and 16 times per day between adolescents and their fathers (*Min* = 1, *Max* = 177). Frequency of proximity between adolescents and parents did not differ between adolescent boys and girls (adolescent–mother: *Mdn*_boys_= 292.5, *Mdn*_girls_ = 369,* z* = −0.935, *p* = .350; adolescent–father: *Mdn*_boys_= 273, *Mdn*_girls_ = 209.5, *z* = −0.757, *p* = .449) and did not correlate with age (adolescent–mother: *r* = −0.14, *p* = .231; adolescent–father: *r =* −0.19, *p* = .124).

#### Duration of proximity

Descriptive information on the duration of time spent in proximity averaged over the two weeks between adolescents and parents is presented in Table 2. Overall, adolescents spent more time in proximity to mothers than fathers throughout the two weeks (paired Wilcoxon’s signed-rank test: *z* = −5.019, *p* < .001). On average, adolescents spent 74.83 minutes per day (*Min* = 4.17, *Max* = 653.23) close to their mothers and 51.02 minutes per day (*Min* = 4.93, *Max* = 563.62) to their fathers).

Duration of proximity between adolescents and parents did not differ between adolescent boys and girls (adolescent–mother: *Mdn*_boys_= 665.51, *Mdn*_girls_ = 913.77,* z* = −0.925, *p* = .355; adolescent–father: *Mdn*_boys_= 553.25, *Mdn*_girls_ = 500.29, *z* = −0.630, *p* = .529) and did not correlate with age (adolescent–mother: *r* = −0.10, *p* = .388; adolescent–father: −0.10, *p* = .417). When assessing weekdays and weekend days separately, adolescents spent on average 65.45 minutes per weekday (*Min* = 4.17, *Max* = 580.04) close to their mothers and 49.85 minutes per weekday (*Min* = 4.93, *Max* = 563.62) to their fathers. Regarding weekends, adolescents spent on average 93.91 minutes per weekend day (*Min* = 5.14, *Max* = 653.23) close to their mothers and 56.55 minutes per weekend day (*Min* = 5.19, *Max* = 542.69) to their fathers. To gain more insight into the average duration of a moment being in proximity, we calculated per individual how long each moment of being in proximity lasted and provided the median. Overall, a moment of being in proximity between adolescents and mothers lasted 19.63 minutes (*Min* = 2.08, *Max* = 320.59), and between adolescents and fathers 16.34 minutes (*Min* = 2.82, *Max* = 229.02). Results on frequency and duration of proximity based on one-sided and combined data are presented in Appendix 5.

### Proximity-triggered questionnaires

#### Adolescents

Proximity data based on BLE beacons were available for 71 adolescents and 66 of these adolescents received proximity-triggered questionnaires. Due to phone settings and technical issues of the method, not all adolescents received these questionnaires. A total of 1620 questionnaires were delivered to these adolescents of which 842 (52.0%) were completed, four were canceled (i.e., the questionnaire was not completed before the expiration time), and 774 expired. On average, 24.55 questionnaires were delivered per adolescent concerning interactions with mothers and fathers (*SD* = 15.68, Min/Max = 2/64). Of the 1620 questionnaires, 913 (56.4%) concerned interactions with mothers, and 707 (44.6%) concerned interactions with fathers. Adolescents fully completed 456 questionnaires on interactions with mothers (49.9%) and 386 questionnaires on interactions with fathers (54.6%) Thus, whether the interactions were with mother or father did not matter for completing or not completing the questionnaires. Regarding the time of day, more questionnaires were not completed during the early morning and more were completed during the evening.

#### Parents

Proximity data based on BLE beacons was available for 139 parents, and 128 of these received proximity-triggered questionnaires. A total of 1937 questionnaires were delivered to these parents of which 974 (50.3%) were completed, six were canceled, and 957 expired. On average, 15.13 questionnaires were delivered per parent (*SD* = 8.75 Min/Max = 1/40).

Detailed descriptive statistics of the subjective quality of the interactions between adolescents and their mothers and fathers are presented in Table 3. In 555 of the 844 answered questionnaires (65.8%) adolescents reported that they had an interaction with their parent (i.e., that they spoke to or with each other). In 793 of the 986 answered questionnaires (80.4%) parents reported that they had an interaction with their adolescent. Overall, adolescents rated the interactions with their parents as rather pleasant, reported high on positive and low on negative affect, and were positive on parental warmth and reported low levels of criticism by both mothers and fathers. A similar pattern of results was found for parental reports.

**Table 3:** Descriptive statistics of experienced quality of interactions for adolescents, mothers, and fathers

	*N*^*a*^	*Obs*	*M*	*SD*	Min	Max
Adolescent report						
Pleasantness interaction mother	49	319	5.66	1.10	1	7
Pleasantness interaction father	50	236	5.56	1.09	1	7
Positive affect interaction mother	49	319	5.65	1.11	1	7
Positive affect interaction father	50	236	5.70	1.05	1	7
Negative affect interaction mother	49	318	1.32	0.67	1	7
Negative affect interaction father	50	236	1.27	0.65	1	7
Parental warmth mother	49	318	5.88	1.09	1	7
Parental warmth father	50	236	5.80	1.21	1	7
Parental criticism mother	49	318	1.56	1.00	1	7
Parental criticism father	50	236	1.53	0.99	1	7
Parent report						
Pleasantness interaction mother	61	472	5.72	1.02	2	7
Pleasantness interaction father	54	319	5.59	1.03	2	7
Positive affect interaction mother	61	472	5.53	1.02	1	7
Positive affect interaction father	54	319	5.47	0.87	1.5	7
Negative affect interaction mother	61	472	1.31	0.69	1	5.33
Negative affect interaction father	54	319	1.32	0.62	1	4.67
Parental warmth mother	61	466	5.88	0.88	1	7
Parental warmth father	54	316	5.61	0.85	3	7
Parental criticism mother	61	466	1.94	1.30	1	7
Parental criticism father	54	315	2.11	1.27	1	6

*Note*. Obs = total number of observations

^a^ Not all parents and adolescents received or completed proximity-triggered questionnaires, therefore *N* is smaller than the sample size

To explore whether frequency and duration of proximity were related to the quality of interactions assessed almost directly after the interactions, we first calculated person-mean scores of the experienced quality. Next, frequency and duration of proximity over the two weeks per dyad were calculated. Subsequently, Pearson correlation analyses were conducted to examine associations between quantity of proximity and quality of the interaction for adolescent–mother, and adolescent–father dyads separately. Results are presented in Table 4. No statistically significant associations were found between frequency of proximity and adolescents’ affect, parents’ affect, and the quality of parenting behavior. Duration of proximity between adolescents and mothers did, however, relate to parental criticism as reported by mothers, with more time of proximity (between adolescents and mothers) being associated with less parental criticism (reported by mothers).

**Table 4:** Correlations of experienced quality of interaction based on proximity-triggered questionnaires and frequency and duration of proximity for adolescent–mother and adolescent–father dyads separately

	1	2	3	4	5	6	7	8	9	10
1. Frequency of proximity		0.94***	−0.08	0.13	−0.12	0.08	−0.14	−0.11	−0.13	−0.17
(n)		(67)	(50)	(50)	(50)	(50)	(54)	(54)	(54)	(54)
2. Duration of proximity	0.92***		−0.17	0.12	−0.06	0.05	−0.08	−0.11	−0.14	−0.18
(n)	(75)		(49)	(49)	(49)	(49)	(54)	(54)	(54)	(54)
3. Positive affect AA	−0.03	−0.10		−0.45***	0.75***	−0.37**	0.44**	−0.46**	0.16	−0.18
(n)	(49)	(49)		(50)	(50)	(50)	(41)	(41)	(41)	(41)
4. Negative affect AA	0.06	0.15	−0.67***		−0.52***	0.48***	−0.47**	0.34*	−0.27	0.07
(n)	(49)	(49)	(49)		(50)	(50)	(41)	(41)	(41)	(41)
5. Parental warmth AP	−0.03	−0.09	0.77***	−0.58***		−0.62***	0.52***	−0.45**	0.24	−0.06
(n)	(49)	(49)	(49)	(49)		(50)	(41)	(41)	(41)	(41)
6. Parental criticism AP	0.11	0.18	−0.50***	0.60***	−0.83***		−0.46**	0.38*	−0.38*	0.20
(n)	(49)	(49)	(49)	(49)	(49)		(41)	(41)	(41)	(41)
7. Positive affect PP	0.24	0.23	0.53***	−0.27	0.38*	−0.13		−0.49***	0.71***	−0.38**
(n)	(61)	(61)	(42)	(42)	(42)	(42)		(54)	(54)	(54)
8. Negative affect PP	−0.24	−0.21	−0.22	0.26	−0.19	0.14	−0.66***		−0.43**	0.49***
(n)	(61)	(61)	(42)	(42)	(42)	(42)	(61)		(54)	(54)
9. Parental warmth PP	0.11	0.13	0.48**	−0.37*	0.42**	−0.30	0.56***	−0.48***		−0.60***
(n)	(61)	(61)	(42)	(42)	(42)	(42)	(61)	(61)		(54)
10. Parental criticism PP	−0.24	−0.28*	−0.48**	0.34*	−0.37*	0.19	−0.51***	0.52***	−0.61***	
(n)	(61)	(61)	(42)	(42)	(42)	(42)	(61)	(61)	(61)	

*Note.* Correlations of adolescent–mother dyads are presented under the diagonal, and correlations of adolescent–father dyads are presented above the diagonal

AA = adolescent about self, AP = adolescent about parent, PP = parent about own behavior

* indicates *p* < .05. ** indicates *p* < .01, *** indicates *p* < .001

A subsequent examination of the associations between all experienced quality measures showed that, in general, adolescents who reported more positive and less negative affect also reported more parental warmth and less parental criticism of mothers and fathers. Interestingly, adolescents’ positive and negative affect were also related to mothers’ parenting behavior reported by mothers, with more positive affect and less negative affect being associated with more (mother self-reported) maternal warmth and less maternal criticism. We did not detect any associations between adolescent affect and fathers’ parenting behavior reported by fathers.

### Sensitivity analyses

By conceptualizing frequency of proximity as number of occurrences of parent–adolescent proximity and duration of proximity as time spent in proximity when proximity was detected in two (or more) consecutive scanning intervals, we expected to capture two levels of parent–adolescent proximity and capture short and longer moments of proximity. However, the very high correlations between frequency and duration (0.94 and 0.92) suggest otherwise. We therefore performed sensitivity analyses using a different conceptualization of frequency of proximity by counting the number of distinct episodes of parent–adolescent proximity (two or more scanning intervals) and conducted Pearson correlation analyses with that variable. The correlations between frequency and duration of proximity were somewhat lower, but still high (0.75 for adolescent–mother proximity and 0.83 for adolescent–father proximity). All other findings remained the same, except that frequency of proximity was positively and significantly related to positive affect of mothers (*r* = 0.27, *p* = .038). See Appendix 6 of the Supplementary Materials for the full table.

## Discussion

The importance of interpersonal relationships and spending time together for well-being has been shown consistently for people in general (e.g., Umberson & Karas Montez, 2010; Sun et al., 2020), and also more specifically for parent–adolescent dyads (Dubas & Gerris, 2002; Desha et al., 2011). These studies mostly have focused on self-report measures providing a rough indication of the amount of time spent together on average or on a daily level. To gain more detailed, objective, and less biased insight into the patterns of being in proximity in the daily flow of life, we introduced a novel method to capture proximity with BLE beacons and a smartphone application. To illustrate this method we assessed the frequency and duration of proximity throughout two weeks for *n* = 145 parent–adolescent dyads. Additionally, we used this proximity to trigger questionnaires to assess the quality of the interactions.

### Tracking proximity between adolescents and parents

Although previous studies have shown that proximity between persons can be tracked using smartphone Bluetooth with or without BLE beacons (e.g., Dlugonski et al., 2019; Ghandeharioun et al., 2016; Van Woudenberg et al., 2020), so far several factors limited the broader and practical use of this method, such as burdening participants with a research phone or selective inclusion of participants with an Android smartphone. To overcome these, the current study combined BLE beacons with a smartphone application that can be installed on *any* smartphone which enables gathering information on proximity between persons in daily life. As an illustration, we applied this method to family life and measured the proximity between adolescents and parents. Analyses on beacon-based proximity measures showed that throughout the two weeks, adolescents were on average more often and longer in proximity to their mothers than to their fathers, which confirms previous research using self-report measures (Larson & Richards, 1991; Phares et al., 2009; Van Lissa & Keizer, 2020). Additionally, we found that adolescents and mothers are on average approximately 75 minutes per day in close proximity, and adolescents and fathers 51 minutes. Although this was more than that found in the previous self-report study in which mothers reported spending approximately 35 minutes per day with their adolescent children and fathers approximately 32 minutes per day (Dubas & Gerris, 2002), we are cautious with interpreting these differences, since not enough information was available to correct for moments that participants were not carrying their smartphones for instance. Our method contributes to a more reliable and ecologically valid assessment, but more research is necessary after further development and validation to for instance adjust for missing or incorrect events of proximity.

The descriptive analyses and visualizations for two families also showed that there is substantial variation between dyads in how often and long adolescents and parents are in proximity. These differences may be a valuable indicator of family cohesion (e.g., enmeshed, normal, disengaged) or quality of the relationship, which could be valuable indices for future studies. Moreover, zooming in to the more detailed level of minutes or hours can yield detailed information on general proximity patterns over the course of a day or week of (sub)groups. Applying this method in large, representative samples could generate normative patterns in parent–adolescent proximity and provide information on how these patterns may change over time. This would also enable further investigation of differences between adolescent–mother, and adolescent–father proximity as well as associations with affect or parenting behavior, as this study may be underpowered due to the sample size. Additionally, the method also provides more information on specific dyads or families. An interesting next step would be to apply multilevel and time series analyses to gain more insight into within-person and within-dyad variation in proximity and elucidate factors that may explain variations, for example, to gain new insight into processes involved in social ruptures and repair.

### Proximity-triggered questionnaires

While being in physical proximity may generally indicate a social interaction (Gupte & Eliassi-Rad, 2012) some adolescents and parents responded sometimes to the first question, on whether they spoke to one another, that this was not the case (20% for parents, 35% for adolescents). This illustrates that being in proximity does not have to imply that there was an actual interaction, as adolescents and parents could for instance be together in the same room, but each be focused on an individual activity, or could be watching television together without actively speaking to each other (i.e., “interacting”). Since the general aim of the RE-PAIR study was to examine parent–adolescent interactions, no follow-up questions were asked if there was no verbal interaction. However, future studies using the method may still want to gain insight into the effect of this shared experience on parent and adolescent mood, for instance. Moreover, we used being in proximity for more than 10 minutes as conceptualization for an interaction, also partly based on technological constraints of scanning behavior of smartphones, but other conceptualizations of interacting could have resulted in different results. With the current conceptualization, shorter interactions have been missed. Additionally, adolescents’ and parents’ indication of not speaking with one another (or not responding to the proximity-triggered questionnaire) may also be due to not wanting to answer questions about a specific interaction (e.g., an emotional or heated interaction), a methodological issue that is difficult to tackle with any method. Despite these considerations, the questionnaires that were answered indicate how valuable it is to combine proximity tracking and triggering questionnaires based on proximity, as it enables gaining more insight into the context of being together.

Another remarkable finding was that we did not find indications for an association between frequency and duration of proximity and adolescent positive and negative affect, whereas previous studies indicated that the quantity of social interactions is related to adolescent well-being (Liu et al., 2019; Sun et al., 2020). These different results may be due to the fact that in these previous studies, interactions were conceptualized differently and were assessed either with the use of EMA (Liu et al., 2019) or by coding audio-recorded snippets of 30 seconds every 10 minutes as interaction or not (Sun et al., 2020). Moreover, these studies focused on all social partners (i.e., friends, romantic partners) rather than parents, as was done in the current study. A pattern that did emerge was that the duration of proximity between adolescents and mothers was related to mothers reporting less parental criticism during interactions. This finding may indicate either that mother–adolescent interactions are shorter in duration if mothers criticize the adolescent (e.g., leading the adolescent to leave the room), or that mothers provide less criticism if mother–adolescent interactions are longer. While replication is necessary, it does provide a first insight into the possible interplay between objectively assessed quantity and experienced quality of parent–adolescent interactions in daily life.

### Limitations, future directions, and implications

Although future work is necessary to further improve methodological aspects, we illustrate that our method is a promising tool to provide a more objective, fine-grained, and ecologically valid assessment of being close to one another. The method could not only be applied to parent–adolescent dyads, but also to track the proximity of the whole family, which is important for adolescent development (Cox & Paley, 1997), or in other contexts (i.e., romantic partners, friends, colleagues). Importantly, we would like to note that the insights provided by our novel method may be impacted by decisions we made and the practical and methodological limitations of the method at this point in time. These may guide future research to further develop the utility of the method. One limitation was that several settings blocked proximity tracking by the Ethica app (e.g., terminating the app or flight mode) and that there were no sufficient data available to correct for this and gain insight into valid recordings and invalid recordings. Although settings on the smartphones were checked by the researchers at the start of the EMA and participants were instructed to not change them, not all participants adhered to these instructions at all times. This may also partly explain the substantial variation in proximity between dyads that we found. Similarly, although we instructed adolescents and parents to take their smartphones and BLE beacons with them at all times, some reported in the evaluation interviews by telephone after the EMA that they left their phones somewhere in the house sometimes or left phones together during the night. We tried to correct this by excluding nighttime from our analyses but did not have sufficient data to correct for all instances. Moreover, due to the rapid technological development of applications and phones, software systems were updated throughout the study which for instance affected the privacy settings of smartphones, disabling the access of the Ethica app to location services in some cases. To reduce the impact of such updates and/or participants’ behavior, we combined information on adolescents’ and parents’ smartphones. To gain more insight into the impact of these settings and support the development and validation of the method, future studies could make use of data donation methods (i.e., asking participants to share the logs of their smartphone) to possibly correct for it.

Additionally, the selection of our beacons, the specific assessments of proximity, and the calculation of the frequency and duration measures may have had an impact on the proximity results. For example, we selected specific BLE beacons that were easy to carry around by adolescents and parents, had a long battery life, and were reusable. Due to the irregular scanning behavior of smartphones, we also specified that an interval between 0 and 7 minutes was acceptable (see Appendix 3 for an explanation of exact cutoffs). Future studies may want to explore and compare the usage of different cutoffs (for duration of proximity) and other types of beacons that may enable detecting proximity with a smaller or larger range. Moreover, as the scanning interval was approximately 5 minutes, more fleeting moments of proximity or interactions were missed and may have resulted in an underestimation of the frequency of being in proximity. Furthermore, we operationalized frequency and duration of proximity as two separate constructs, but they were highly correlated and the utility of one or both constructs could be addressed in future studies. With regard to proximity-triggered questionnaires, we first decided on an expiration period of 10 but later on 30 minutes to ensure that adolescents and parents were no longer in each other proximity and independently could complete the questionnaires. However, this may have resulted in participants missing the survey due to being on the road or being busy with other tasks. To reduce the burden on participants, the proximity-triggered questionnaires were blocked for 4 hours after a trigger, since the method was implemented in a larger study, which also included an EMA protocol with a semirandom schedule. Future studies may want to solely use these proximity-triggered questionnaires to gain a more representative idea of the context and content of interactions in daily life.

In addition to the potential applications within the scientific domain, the method could also be used as a diagnostic and intervention tool in clinical practice. Both the proximity-tracking and proximity-triggered surveys have the potential to contribute to providing valuable and tailored feedback in clinical settings. Proximity tracking could help in mapping the social network and interactions of a person, couple, or family in treatment and may indicate when time spent in proximity is remarkably low (or high). It can be insightful for treatment (such as system therapy or couples therapy) to relate this information to how the person experiences these moments of social proximity and potentially the interactions that follow or do not follow. Moreover, a next step could involve sending personalized proximity-triggered messages, for instance by sending parents a message on how to express empathy or warmth when in proximity of their child with mental health problems, or encouragement to seek contact after a period with no interaction in couples. Here the possibilities are endless and may be highly valuable in helping clients realize certain treatment goals.

## Conclusion

Research has indicated the importance of assessing proximity between persons, since people generally feel happier and more connected when they spend more time with others. Whereas the use of self-report questionnaires has enhanced our understanding, objective information on behavioral patterns of proximity between individuals may generate important new insights. We present a novel method with BLE beacons and a smartphone application to assess proximity between persons and illustrate this with data on *n* = 145 parent–adolescent dyads. We show that unobtrusively tracking proximity between adolescents and parents can yield valuable insights into the frequency and duration of being in proximity. By also triggering questionnaires based on this proximity to assess the quality of parent–adolescent interaction in daily life, this method is a promising tool that could contribute to quantifying social behaviors and enhance the understanding of social interactions in daily life and in clinical practice.

### Supplementary Information

Below is the link to the electronic supplementary material.

Supplementary Materials (PDF 175 KB)
