# Supplementary material for: Tracking real-time proximity in daily life: A new tool to examine social interactions
Source: Behav Res Methods. 2024 Apr 29;56(7):7482–97. doi: 10.3758/s13428-024-02432-1 (PMC11362181; doi:10.3758/s13428-024-02432-1)
Supplement: Supplementary file 1 — Supplementary Materials (PDF 175 KB) [file 13428_2024_2432_MOESM1_ESM.pdf]

Supplementary Materials

Tracking real-time proximity in daily life: A new tool to examine social interactions

Behavior Research Methods

Loes H. C. Janssen[1,2], Bart Verkuil[1,2], Andre Nedderhoff[1], Lisanne A.E.M. van Houtum[1,2], Mirjam C.M. Wever[1,2], & Bernet M. Elzinga[1,2]

[1]*Department of Clinical Psychology, Leiden University, Leiden, the Netherlands;*

[2]*Leiden Institute for Brain and Cognition (LIBC), Leiden, the Netherlands*

Contact corresponding author l.h.c.janssen@fsw.leidenuniv.nl

Appendix 1.

Detailed information of missing data.

After cleaning the data, inspection of the descriptive overview indicated missing data. For three families there was no available data from both sides (i.e., adolescent to parent and parent to adolescent) either due to errors in the settings of the BLE beacons or due to non-working BLE beacons. In one of these three family, there was data of the smartphone of the mother detecting the BLE beacon of the father and vice versa, but since the current study focused on parent-adolescent proximity, data of the full family was removed from the dataset. This resulted in available data of 77 families. In some cases, only one-sided data was present. The Ethica app on the smartphones of 11 participants (i.e., six adolescents, two mothers, and three fathers) did not detect any BLE beacons of other family members in close proximity. This was most likely due to errors in the smartphone or Ethica app settings of participants (i.e., not providing or retracting permission to access location services). Moreover, the Ethica app of one father detected BLE beacons in close proximity only outside the participation period. Thus, proximity data based on detecting BLE beacon by the Ethica app on the smartphone was missing for a total of twelve participants. Concerning proximity based on BLE beacon of a participant detected by the Ethica app of family members, data of eight participants (i.e., two adolescents, five mothers, and one father) was missing. This was most likely caused by the BLE beacon not working properly or incorrect settings.

Several factors could result into missing data, related to the working of the BLE beacons or the Ethica app. Although BLE beacons that were returned by families were checked for defects and battery life before reusing, some BLE beacons seemed to be broken since they could not be detected anymore. These were no longer used in the study. Several settings on the smartphone, as mentioned in the manuscript, could impact the scanning process by the Ethica app. Terminating the app or switching off Bluetooth was detected by Ethica and participants would receive an error message of the app which would require them to reopen the app or turn Bluetooth back on. How often this message was shown was not logged. During the instruction session, we stressed the importance of not changing settings and not using specific modes but were unable to track these settings consistently throughout the study.

There is not sufficient information available to provide a sense of how often above described settings impacted the scanning process and possibly caused missing data. The Ethica app could not track general phone settings such as flight and night mode that affect beacon tracking. While the Ethica app did detect and log when Bluetooth was switched on and off, the log data is messy with also 'unknown' changes in settings. Moreover, the Ethica app also detected when the application was opened and closed, however, these logs also contain a lot of noise as opening and closing of the app to complete questionnaires is also logged.

Appendix 2.

Rationale for specified time interval used to indicate adolescents' and parents' smartphones detecting each other's BLE beacon around the same time.

Since data on proximity was collected independently per smartphone, a differentiation was made between detection of proximity by one smartphone (either the adolescent or parent) and detection of proximity by both smartphones (adolescent and parent) around the same time point. To specify a time interval to indicate connection to each other around the same time, we used the full cleaned dataset (including data on proximity between adolescents and parents and between parents during the day and night).

First, the dataset was split per individual (adolescent, mother, and father) and all connections of an individual's smartphone with its own BLE beacon were removed from the data. Next, every row of information was labeled to indicate to whom the connection was (i.e., adolescent to mother, adolescent to father, mother to adolescent, mother to father, father to adolescent, and father to mother) and afterwards the files were merged again. Subsequently, we matched the data of the individuals per connection type based on day and time and calculated the time difference. For example, if the smartphone of adolescent detected mother's BLE beacon on day 1 at 10.01 AM and mother's smartphone detected adolescent's BLE beacon on day 1 at 10.02 AM, a time difference of 1 minute was calculated.

Descriptive statistics of these time differences in minutes were inspected to decide a cut-off for detection of proximity by both the adolescent and parent. The distribution was highly skewed (Figure A2.1), with a median value of 2.46 minutes (Min = 0, Max = 111120). This suggests that 2.5 minutes could be used as a cut-off time interval to indicate adolescents' and parents' smartphones detecting each other's BLE beacon around the same time. This data-driven suggestion can be substantiated by the scanning interval of smartphones. This interval should be approximately 5 minutes and it is not expected that smartphones of family members scan in sync. Taking the half of 5 minutes, 2.5 minutes seems reasonable and aligns with the median found in the data. Thus, we decided to use 2.5 minutes as time difference cut-off. That is, if time differences between scans of individuals were less than 2.5 minutes,

it indicated adolescents and parents being in proximity around the same time (labeled ‘both connected’). If time intervals were larger, it was labeled as ‘one connected’.

It should be noted that the distribution in Figure A2.1 may be a bit biased since it includes data from each individual perspective (adolescent’s smartphone detected mother’s BLE beacon on day 1 at 10.01 AM and mother’s smartphone detected adolescent’s BLE beacon on day 1 at 10.02 AM results in a 1 minute time difference and mother’s smartphone detected adolescent’s BLE beacon on day 1 at 10.02 AM and adolescent’s smartphone detected mother’s BLE beacon on day 1 at 10.01 AM results in a 1 minute time difference).

As the scanning interval of smartphones approximates 5 minutes and can differ per smartphone brand (see for more information Appendix 3), we checked how this impacted the data the label ‘both connected’. That is, we compared how many data points were labeled as both connected using adolescents’ data as the basis and how many data points were labeled as both connected using parents’ data as the basis. Results showed some differences. Overall, adolescents’ smartphones connected 11407 times to mothers’ BLE beacon around the same time. Mothers’ smartphones connected 12028 times to adolescents’ BLE beacon around the same time. Adolescents’ smartphones connected 7724 times to fathers’ BLE beacon around the same time. Fathers’ smartphones connected 9142 times to adolescents’ BLE beacon. These differences might be due to for instance 1 minute time intervals which caused that multiple time points of parents’ smartphone connecting to adolescents’ BLE beacon were linked to one time point of adolescents’ smartphone connecting to parents’ BLE beacon. We decided to use data labeled as both connected from adolescents’ perspective throughout the paper, as this is the most restricted.

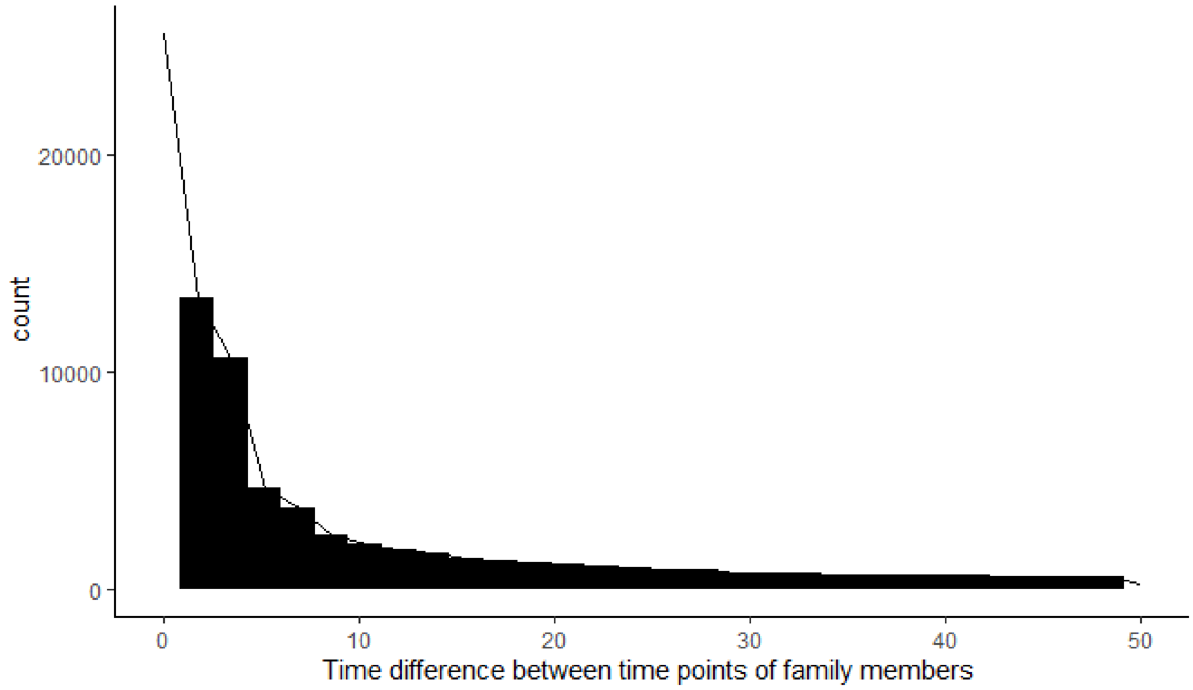

Figure A2.1. Distribution of time differences (< 50 minutes) between data of dyads being in proximity of the full sample

Appendix 3.

Rationale for specified scanning interval cut-off.

To gain more insight into the range and distribution of time intervals between scans, we calculated the time (in minutes) between each scan per individual and The distribution of time intervals between scans was skewed (Figure A3.1) with a median value of 5 minutes (Min = 0, Max = 12445). When zooming in to the distribution (Figure A3.2) we noticed two things: 1) there was substantial variation around the 5 minute interval, and 2) there was a large number of 1-minute intervals. We further investigated these 1-minute intervals and checked whether intervals below 2 minutes were related to brand of the smartphone. Figure A3.3 shows that intervals below 2 minutes were more common for certain brands (e.g., iPhones). Since duration of proximity was calculated by summing time intervals between two (or more) consecutive scans, these 1-minute intervals could bias the results.

We therefore counted the occurrence of two (or more) consecutive 1-minute intervals and this was the case for 30 participants (13.5%). For 21 participants, there was one occurrence of two consecutive 1-minute intervals and for three participants this occurred twice. For the other six participants, either two consecutive 1 minute intervals occurred more often (Max = 27 times) and/or more than two consecutive 1-minute intervals occurred (Max = 40 consecutive time intervals). Based on these descriptive statistics it was decided to include the 1-minute time intervals. As a maximum value for scanning intervals we decided to use 7 minutes, based on visual inspection of Figure A3.2. That is, if two (or more) consecutive scanning intervals were between 0 and 7 minutes, then the intervals were used to calculate the duration of proximity.

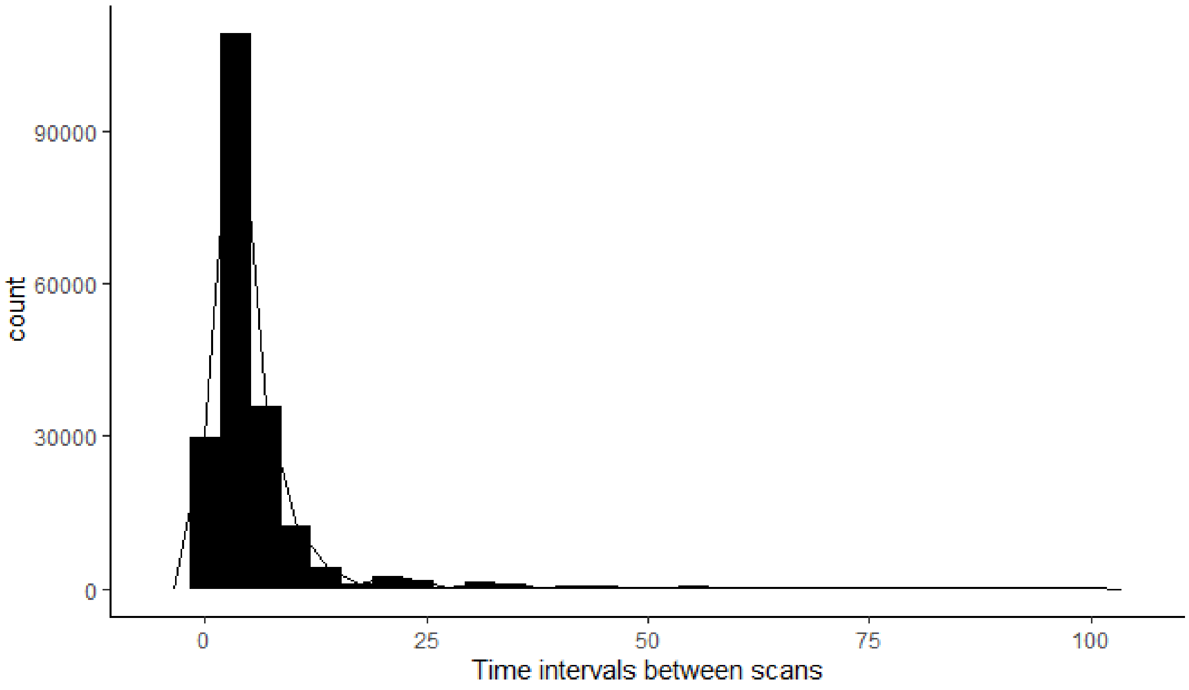

Figure A3.1. Distribution of time intervals between scans (< 100 minutes) of individuals of the full sample

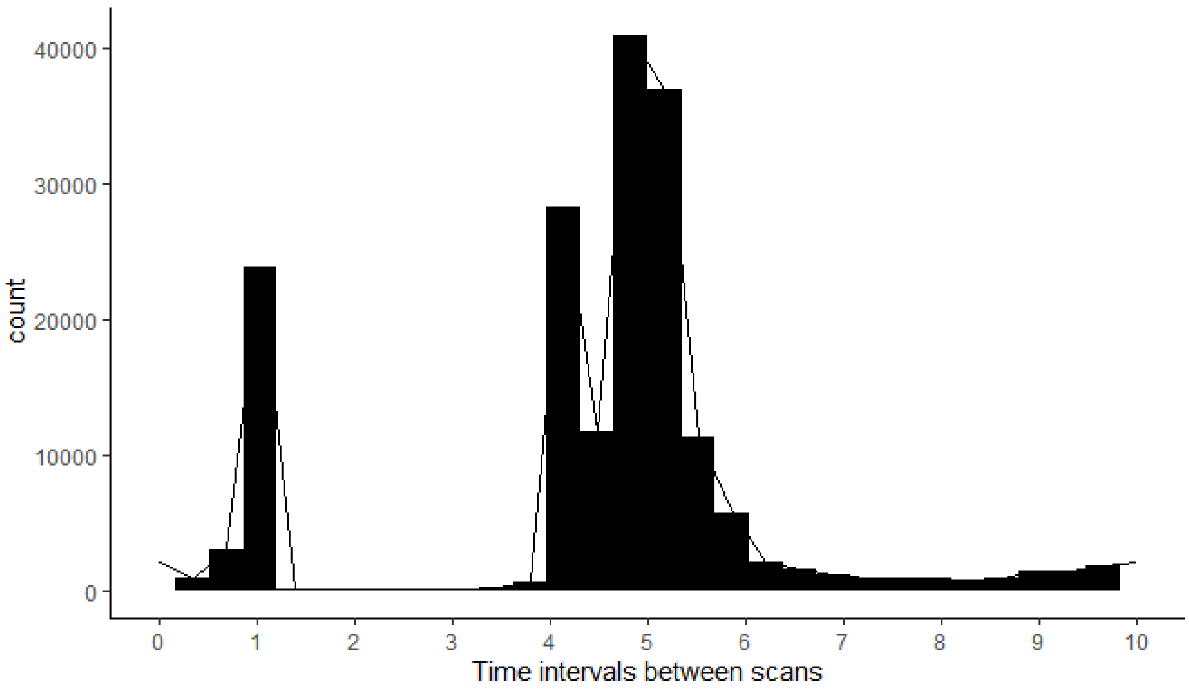

Figure A3.2. Distribution of time intervals between scans (< 10 minutes) of individuals of the full sample

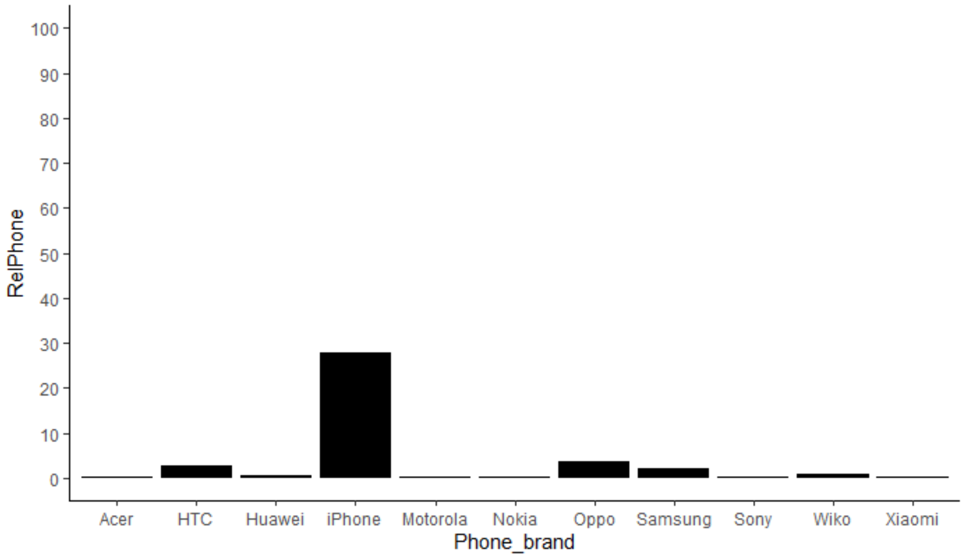

Figure A3.3. Proportion how often two minute intervals occurred per phone brand.

Appendix 4.

Rationale for specified nighttime.

In the first questionnaires of the day of RE-PAIR, which participants received at 7AM on weekdays (Monday until Friday) and 9AM on weekend days (Saturday and Sunday), adolescents and parents indicated the time they went to bed and the time they got up. We first selected EMA data of the 77 adolescents and 145 parents of whom proximity data was available. Next, we calculated the average time of going to bed and getting up for parents and adolescents for week and weekend days separately. The results are presented below in Table A4.1. Despite substantial individual differences, the average times were used and we excluded data during the week collected between 9.30PM to 7.00AM and between 23PM to 9PM during the weekend.

Several factors may have influenced the calculated average, but we did not correct for these since this information was not consistently available of all participants. Some adolescents or parents worked night shifts (i.e., health care or police work) and were not able to answer the morning questionnaire or reported on those days that they for instance went to bed at 4PM. Leaving the questions about sleep unanswered in the morning was not possible. This may have led to a bias of the rise- and bedtime. Furthermore, providing a time for going to bed or getting up was impaired for a couple of days for some participants due to an update of the Ethica app. In these cases, participants were not able to change the time which resulted in the time of going to bed and getting up being exactly the same and corresponding to the time of receiving the survey.

Table A4.1

Average time of getting up and going to bed indicated by adolescents and parents

	Adolescents (*n/obs)*	Parents (*n/obs)*
Weekday		
Risetime	7.14 AM (76/555)	6.49 AM (145/555)
Bedtime	9.34 PM (76/555)	10.57 PM (145/555)
Weekend day		
Risetime	8.57 AM (72/203)	8.23 AM (145/1283)
Bedtime	10.45 PM (72/203)	11.37 PM (145/496)

Appendix 5.

Frequency and duration of proximity per individual and combined.

Since proximity data collection was two-sided, we calculated the frequency and duration of proximity for each individual separately. That is, adolescent in proximity of mother, adolescent in proximity of father, mother in proximity of adolescent, and father in proximity of adolescent. Results are provided in Table A6.1. Additionally, frequency and duration of proximity was calculated for the most conservative measure of proximity (i.e., smartphones of both the adolescent and parent detected each other's BLE beacon around the same time).

Although these descriptive statistics indicated that scanning behavior of the smartphones and the use of smartphones by the participants had a substantial impact on both the frequency and duration of being in proximity, the main findings that adolescents were more often and longer in proximity of mothers than fathers remained significant.

Table A6.1

Descriptive statistics of the frequency and duration of adolescent-parent proximity during the two weeks

					Difference test	
	N	*Mdn*	Min	Max	*z*	*p*
Frequency						
Adolescent → mother	64	208	2	852		
Adolescent → father	62	146	9	885	-3.911[a]	< .001
Mother → adolescent	71	237	1	1174		
Father → adolescent	62	148.5	14	1029	-2.986[b]	.002
Adolescent ←→ mother	56	89	1	660		
Adolescent ←→ father	56	53.5	1	400	-3.031[a]	.002
Duration						
Adolescent → mother	63	564.15	25	3694.64		
Adolescent → father	60	405.55	9.97	3677.11	-3.709[a]	< .001
Mother → adolescent	67	623.9	21.11	3705.79		
Father → adolescent	62	338.81	12.88	2946.12	-3.726[b]	< .001
Adolescent ←→ mother	56	262.32	6.02	2789.85		
Adolescent ←→ father	52	174.58	5	1359.49	-2.992[a]	.003

Note. The median was reported since frequency and duration of close proximity between adolescents and parents was non-normally distributed (all p's < .001).

[a]Paired Wilcoxon's signed rank test was used to test difference between adolescent → mother and adolescent → father frequency (n = 56) and duration (n = 53), and adolescent ←→ mother and adolescent ←→ father frequency (n = 45) and duration (n = 43)

[b]Unpaired two-samples Wilcoxon test was used to test difference between mother-adolescent and father-adolescent frequency (n = 133) and duration (n = 129)

Appendix 6.

Correlations of experienced quality of interaction based on proximity triggered questionnaires and frequency and duration of proximity for adolescent-mother and adolescent-father dyads separately

	1.	2	3	4	5	6	7	8	9	10
1. Frequency of proximity (*n*)		0.83*** (67)	0.02 (50)	0.11 (50)	-0.13 (50)	0.13 (50)	-0.13 (54)	-0.13 (54)	-0.15 (54)	-0.09 (54)
2. Duration of proximity (*n*)	0.75*** (75)		-0.17 (49)	0.12 (49)	-0.06 (49)	0.05 (49)	-0.08 (54)	-0.11 (54)	-0.14 (54)	-0.18 (54)
3. Positive affect AA (*n*)	0.02 (49)	-0.10 (49)		-0.45*** (50)	0.75*** (50)	-0.37** (50)	0.44** (41)	-0.46** (41)	0.16 (41)	-0.18 (41)
4. Negative affect AA (*n*)	0.08 (49)	0.15 (49)	-0.67*** (49)		-0.52*** (50)	0.48*** (50)	-0.47** (41)	0.34* (41)	-0.27 (41)	0.07 (41)
5. Parental warmth AP (*n*)	-0.06 (49)	-0.09 (49)	0.77*** (49)	-0.58*** (49)		-0.62*** (50)	0.52*** (41)	-0.45** (41)	0.24 (41)	-0.06 (41)
6. Parental criticism AP (*n*)	0.19 (49)	0.18 (49)	-0.50*** (49)	0.60*** (49)	-0.83*** (49)		-0.46** (41)	0.38* (41)	-0.38* (41)	0.20 (41)
7. Positive affect PP (*n*)	0.27* (61)	0.23 (61)	0.53*** (42)	-0.27 (42)	0.38* (42)	-0.13 (42)		-0.49*** (54)	0.71*** (54)	-0.38** (54)
8. Negative affect PP (*n*)	-0.17 (61)	-0.21 (61)	-0.22 (42)	0.26 (42)	-0.19 (42)	0.14 (42)	-0.66*** (61)		-0.43** (54)	0.49*** (54)
9. Parental warmth PP (*n*)	0.08 (61)	0.13 (61)	0.48** (42)	-0.37* (42)	0.42** (42)	-0.30 (42)	0.56*** (61)	-0.48*** (61)		-0.60*** (54)
10. Parental criticism PP (*n*)	-0.22 (61)	-0.28* (61)	-0.48** (42)	0.34* (42)	-0.37* (42)	0.19 (42)	-0.51*** (61)	0.52*** (61)	-0.61*** (61)	

Note. Correlations adolescent-mother dyads are presented under the diagonal, correlations adolescent-father dyads are presented above the diagonal.
AA = adolescent about self, AP = adolescent about parent, PP = parent about own behavior
* indicates $p < .05$. ** indicates $p < .01$, *** indicates $p < .001$.
